# Subjective brain fog: a four-dimensional characterization in 25,796 participants

**DOI:** 10.3389/fnhum.2024.1409250

**Published:** 2024-06-06

**Authors:** Ali Alim-Marvasti, Matteo Ciocca, Narayan Kuleindiren, Aaron Lin, Hamzah Selim, Mohammad Mahmud

**Affiliations:** ^1^UCL Queen Square Institute of Neurology, University College London, London, United Kingdom; ^2^Research Division, Mindset Technologies Ltd., London, United Kingdom; ^3^Department of Brain Sciences, Imperial College London, London, United Kingdom

**Keywords:** brain fog, cognition, long COVID, functional cognitive disorder, MCI (mild cognitive impairment)

## Abstract

**Importance:**

Brain fog is associated with significant morbidity and reduced productivity and gained increasing attention after COVID-19. However, this subjective state has not been systematically characterised.

**Objective:**

To characterise self-reported brain fog.

**Design:**

We systematically studied the cross-sectional associations between 29 *a priori* variables with the presence of “brain fog.” The variables were grouped into four categories: demographics, symptoms and functional impairments, comorbidities and potential risk factors (including lifestyle factors), and cognitive score. Univariate methods determined the correlates of brain fog, with long-COVID and non-long-COVID subgroups. XGBoost machine learning model retrospectively characterised subjective brain fog. Bonferroni-corrected statistical significance was set at 5%.

**Setting:**

Digital application for remote data collection.

**Participants:**

25,796 individuals over the age of 18 who downloaded and completed the application.

**Results:**

7,280 of 25,796 individuals (28.2%) reported experiencing brain fog, who were generally older (mean brain fog 35.7 ± 11.9 years vs. 32.8 ± 11.6 years, *p* < 0.0001) and more likely to be female (OR = 1.2, *p* < 0.001). Associated symptoms and functional impairments included d*ifficulty focusing or concentrating* (OR = 3.3), *feeling irritable* (OR = 1.6), *difficulty relaxing* (OR = 1.2, all *p* < 0.0001), *difficulty following conversations* (OR = 2.2), *remembering appointments* (OR = 1.9), *completing paperwork* and performing *mental arithmetic* (ORs = 1.8, all *p* < 0.0001). Comorbidities included long-COVID-19 (OR = 3.8, *p* < 0.0001), concussions (OR = 2.4, *p* < 0.0001), and higher migraine disability assessment scores (MIDAS) (+34.1%, all *p* < 0.0001). Cognitive scores were marginally lower with brain fog (−0.1 std., *p* < 0.001). XGBoost achieved a training accuracy of 85% with cross-validated accuracy of 74%, and the features most predictive of brain fog in the model were difficulty focusing and following conversations, long-COVID, and severity of migraines.

**Conclusions and relevance:**

This is the largest study characterising subjective brain fog as an impairment of concentration associated with functional impairments in activities of daily living. Brain fog was particularly associated with a history of long-COVID-19, migraines, concussion, and with 0.1 standard deviations lower cognitive scores, especially on modified Stroop testing, suggesting impairments in the ability to inhibit cognitive interference. Further prospective studies in unselected brain fog sufferers should explore the full spectrum of brain fog symptoms to differentiate it from its associated conditions.

## Highlights

- **Question:** What are the correlates of subjective brain fog as a syndrome, and can it be homogenously characterised?
- **Findings:** In this large study of 25,796 participants, self-defined brain fog was well characterised using the four dimensions of demographics (females, older mean age 35.7 ± 11.9 years), symptoms and functional impairments (difficulty focusing, concentrating, following conversations, remembering appointments), comorbidities (mainly COVID-19, migraines, concussions, but also anxiety, depression and poor sleep), and objective cognitive function (0.1 standard deviations worse, with the largest deficit in cognitive flexibility and ability to inhibit cognitive interference: 0.13 standard deviations worse, *p* < 0.0001). No problems concentrating stratified a minority of individuals with brain fog into a less functionally impaired group.
- **Meaning:** Brain fog sufferers exhibited surprisingly homogenous symptoms and impairments with significant overlap with migraine disability scores. Brain fog symptoms should be explored by taking a migraine, COVID-19, and concussion history and addressing a combination of treatable comorbidities and lifestyle factors.

## Introduction

The nebulous symptom of brain fog has recently gained increasing attention, particularly in the context of long-COVID-19 and its associated prolonged sequelae involving cognitive, social, occupational, and mental health morbidity (Díez-Cirarda et al., 2023; Taquet et al., 2023). This surge in interest has been in the hope to define the condition, identify its causes, and find treatments (Guo et al., 2022; Jarrott et al., 2022; Spudich and Nath, 2022). Brain fog is associated with reduced cognitive function and can have a crippling effect on daily functioning, time off work, and quality of life especially when associated with long-COVID-19 (Chatys-Bogacka et al., 2022; Liu et al., 2022; Lam et al., 2023). Nevertheless, brain fog has not been adequately characterised, and it is therefore unclear how heterogenous a subjective syndrome it may be (Jennings et al., 2022; Nouraeinejad, 2022).

Studies have suggested COVID-19 is associated with reduced objective cognitive performance, and it has been assumed that this is an objective measure of the brain fog reported by long-COVID-19 patients (Hampshire et al., 2021; Guo et al., 2022). However, it is well known that long-COVID-19 sufferers report “good” and “bad” days without these subjective states correlating with objective measures of cognition (Guo et al., 2022) and that brain fog is associated with a host of non-cognitive somatic symptoms (Jennings et al., 2022). Therefore, brain fog is unlikely to be adequately captured by objective measures alone.

When experiencing the raw symptoms of brain fog, individuals generate internal cognitive and emotional representations of their symptoms that become entangled with the symptoms themselves. Cognitive representations include the identity of the illness (the symptoms that are experienced and the “brain fog” label assigned to them), beliefs about the aetiology and immediate consequences of their brain fog on their daily life, long-term prognosis about duration of symptoms and outcomes, and factors that they believe can control or cure their brain fog (Leventhal et al., 2020). Cognitive and emotional representations can influence both behaviour and outcomes, such that interventions on illness perceptions can improve outcomes in diverse conditions including even diabetes and heart disease (Petrie et al., 2002).

Therefore, assessing the cognitive representation of illness, especially for a syndrome such as brain fog that is primarily perceived and evaluated as affecting cognitive function, requires probing into its internal representations (Weinman et al., 1996; Broadbent et al., 2006).

The purpose of this study was therefore to determine the characteristics and heterogeneity of brain fog as a syndrome in a very large group of individuals, irrespective of whether they had long-COVID-19 or not.

## Methods

### Setting: smartphone application

The data was acquired from a smartphone application named Mindstep which contains questionnaires that gather background medical information (Rifkin-Zybutz et al., 2021), evaluate cognitive function (Alim-Marvasti et al., 2022a,b), evaluate severity of anxiety, depression (Kuleindiren et al., 2022), and post-concussive symptoms (Alim-Marvasti et al., 2022a,b). An application update, released in September 2022, routinely asked whether users experienced self-defined “brain fog.” The application was available to iPhone users in the Apple Application Store and has been previously validated (Rifkin-Zybutz et al., 2021).

### Ethical approval

On using the application users agreed to transparent terms and conditions which included having their data stored and anonymously used for further research. Data from this application was used alongside our data pertaining to in-person questionnaires within the ethics framework approved by West Midlands research ethics committee. All data were stored in line with GDPR regulations.

### Participants

Participants constituted a convenience sample of consecutive data obtained from individuals over the age of 18 who, of their own accord, downloaded and completed the Mindstep application between the 15th of September and the 18th of November 2022. Participants were not actively recruited nor paid. Participants were therefore a convenience sample looking to optimise their brain or mental health by downloading the application. Individuals were not specifically targeted for brain fog. As the sample was all participants who completed the application, there was no missing data.

Participants gave informed consent for the collection and use of their anonymised data for research, as previously described (Rifkin-Zybutz et al., 2021; Kuleindiren et al., 2022; Alim-Marvasti et al., 2022a,b).

### Data

The exact wording of the question on brain fog was: “Have you experienced, or been diagnosed with any of the following (select all that apply).” Options included: Headache or migraine, concussion, Brain fog, Long COVID, Dizziness, None apply to me.

The list of the other 29 *a priori* variables selected for this study are shown in Table 1. Other routinely collected data by the application that are not the focus of this study and therefore were not analysed, have been previously described (Rifkin-Zybutz et al., 2021; Kuleindiren et al., 2022). The application routinely collected data on participant’s age, sex, presence of comorbidities, severity of anxiety and depression symptoms (Kuleindiren et al., 2022), lifestyle habits including diet and self-assessed sleep quality, and evaluated cognitive scores (Alim-Marvasti et al., 2022a,b).

**Table 1:** Studied variables.

The 29 variables	Total *n* = 25,796	Brain fog *n* = 7,280	No brain fog *n* = 18,516	Effect size (odds ratio [CI], % change)	*p*-values
Binary variables					
Long COVID-19	**5.0%**	**10.4%**	**2.9%**	**3.8 [3.2, 4.6]**	**<0.0001**
Difficulty focusing/concentrating	**86.4%**	**94.3%**	**83.3%**	**3.3 [2.8, 3.9]**	**<0.0001**
Concussion	**2.2%**	**3.8%**	**1.6%**	**2.4 [1.9, 3.2]**	**<0.0001**
Difficulty following conversations	**47.8%**	**61.8%**	**42.3%**	**2.2 [2.0, 2.4]**	**<0.0001**
Difficulty remembering appointments	**37.7%**	**48.9%**	**33.3%**	**1.9 [1.8, 2.1]**	**<0.0001**
Difficulty with paperwork	**36.8%**	**46.8%**	**32.9%**	**1.8 [1.6, 2.0]**	**<0.0001**
Difficulty with arithmetic	**29.7%**	**39.1%**	**25.9%**	**1.8 [1.7, 2.0]**	**<0.0001**
Difficulty planning ahead	**47.5%**	**57.0%**	**43.7%**	**1.7 [1.6, 1.9]**	**<0.0001**
Difficulty driving	**10.1%**	**13.5%**	**8.8%**	**1.6 [1.4, 1.8]**	**<0.0001**
Irritable	**85.0%**	**88.9%**	**83.5%**	**1.6 [1.4, 1.8]**	**<0.0001**
Feeling Bad	**76.2%**	**80.1%**	**74.6%**	**1.4 [1.2, 1.5]**	**<0.0001**
Difficulty Relaxing	**76.9%**	**79.2%**	**76.1%**	**1.2 [1.1, 1.3]**	**<0.0001**
Female	**89.2%**	**90.4%**	**88.8%**	**1.2 [1.0, 1.4]**	**<0.001**
Oily fish	**15.1%**	**16.3%**	**14.6%**	**1.1 [1.0, 1.3]**	**<0.001**
Nuts & seeds	**28.5%**	**30.5%**	**27.8%**	**1.1 [1.0, 1.3]**	**<0.0001**
Epilepsy	1.1%	1.2%	1.1%	1.0 [0.7, 1.6]	0.77
Alcohol	57.0%	56.0%	57.4%	0.9 [0.9, 1.0]	0.04
Greens	56.1%	56.7%	55.9%	1.0 [0.9, 1.1]	0.25
Fast Food	41.8%	43.0%	41.4%	1.1 [1.0, 1.2]	0.019
Fruits	28.9%	29.0%	28.9%	1.0 [0.9, 1.1]	0.91
Continuous variables (± standard deviations)					
MIDAS	**10.6 ± 9.1**	**12.2 ± 9.4**	**9.1 ± 8.4**	**+ 34.1%**	**<0.0001**
Patient health questionnaire depression score (PHQ-9)^2^	**13.5 ± 5.1**	**14.3 ± 5.0**	**13.1 ± 5.1**	**+ 9.2%**	**<0.0001**
Age (years) mean ± std Median [IQR] Range	**33.6 ± 11.8** **31 [24, 42]** **[18, 84]**	**35.7 ± 11.9** **34 [25, 45]** **[18, 81]**	**32.8 ± 11.6** **30 [23, 40]** **[18, 84]**	**+ 8.8%**	**<0.0001**
Sleep quality		**4.1 ± 2.3**	**4.4 ± 2.3**	**- 6.8%**	**<0.0001**
Generalised anxiety disorder assessment score (GAD-7)	**11.7 ± 4.9**	**12.2 ± 4.8**	**11.5 ± 4.9**	**+ 6.1%**	**<0.0001**
M-CogScore (z-score)	**0.00 ± 0.72**	**−0.07 ± 0.73**	**0.03 ± 0.71**	**−0.1 std**	**<0.0001**
Smoking pack-years	0.035 ± 0.120	0.037 ± 0.126	0.035 ± 0.117	+ 5.7%	0.76
Ordinal variables					
Exercise (Frequency and Intensity)	**0.22 ± 0.28**	**0.21 ± 0.27** **0.067** **IQR [0, 0.33]**	**0.22 ± 0.28** **0.067** **IQR [0, 0.4]**	**- 4.5%**	**<0.001**
Education	0.69 ± 0.32	0.62 ± 0.32	0.68 ± 0.32		0.15

Variables of interest and data types. Sorted by data type, significance, and effect size. GAD-7: Generalised Anxiety Disorder Questionnaire. PHQ-9: Patient Health Questionnaire. Bold = significant after Bonferroni correction. Std = standard deviation.

Data was fully anonymised and stored in secure Amazon web services cloud.

The *a priori* variables were collected as part of the following four categories:

- Demographics: age, sex, and highest level of education. Education was ordinal with the following levels: no education, secondary school, A-levels, and university degree or higher.
- Symptoms and functional impairments (binary variables): difficulty focusing or concentrating, difficulty relaxing, feeling irritable or bad. Functional questions included difficulty completing paperwork, planning ahead, mental arithmetic, driving, following conversations, and remembering appointments.
- Comorbidities and potential risk factors (including lifestyle factors): migraine disability assessment score (MIDAS), sleep quality scale (SQS) (Snyder et al., 2018), anxiety (GAD-7), depression (PHQ-9) (Kuleindiren et al., 2022), previous diagnosis of long COVID-19 (binary), diagnosis of epilepsy (binary), and whether they had any diagnosed concussions (binary). Questions were asked as such “Have you had a diagnosis of long COVID-19?” or “Have you ever had a diagnosed concussion?” {Alim-Marvasti et al., 2022b, #1893}.
  Lifestyle factors were excessive alcohol intake more than 14 units per week, smoking pack-years, and dietary intake including fruits, oily fish, nuts and seeds, fast food, and greens appearing regularly at least three times a week in their regular diet (binarized). Exercise combined a score of self-reported intensity of exercise with frequency of exercise for an ordinal score.
  The MIDAS score determines the number of missed days and reduced productivity from migraines in three areas: work or school, house chores, and leisure /social activities and has been extensively validated. We used the standard first 5 questions in the MIDAS questionnaire (Stewart et al., 1999; Lipton et al., 2001; Bigal et al., 2003).
  The SQS score is a self-reported single-item sleep scale from 0 to 10, with 0 on the scale being described as “terrible,” 1–3 as “poor,” 4–6 as “fair,” 7 through to 9 as “good,” and 10 as “excellent” (Snyder et al., 2018).
- Cognitive scores were evaluated using the smartphone-based M-CogScore which combines measures of delayed recall, Stroop, and digit symbols modality test with speed of test completion (Alim-Marvasti et al., 2022a,b). As the exact tests have been described in detail previously, we briefly summarise the tests here.

### Cognitive testing

The M-CogScore involved 3 tests. The first test, named M-Memory, was scored using the total number of correctly remembered words out of eight, over two consecutive presentations. Each word flashed for 1 s followed by cued recall, before a second presentation of the same eight words again each flashing for 1 s. The second and third M-CogScore subsections were then tested before a delayed recall for the final M-Memory score. During delayed recall, there were eight multiple choices for each correct word, i.e., seven were distractors.

Between the second presentation and delayed recall in M-Memory, the M-Stroop test was presented. This involved 18 trials of the coloured texts either “red,” “green,” or “blue,” with the participant being presented with a list of three colours to choose the correct answer from. The colours and text were incongruent in the majority of cases (12 trials) and congruent in the rest (6 trials). The M-Stroop score was the total number of correct answers divided by the time taken, combining accuracy and speed.

The third test was M-Symbols. This involved participants writing the answers with their fingers on the smartphone screen, by matching nine symbols to their corresponding numbers using the displayed key. A custom neural network automatically identified the written answers and compared to the correct answers. M-Symbols scores were therefore the number of symbols correct divided by the time taken to complete the test.

Further details on the tests and aspect of cognition that they measure can be found in our original paper (Alim-Marvasti et al., 2022a,b). The only difference from the methodology described previously to validate this score was that the scores were Z-scored on this larger cohort of 25,796 individuals.

### Data sharing statement

Anonymised analysis-ready data and the statistical analyses are available upon reasonable request by contacting the corresponding author.

### Univariate and subgroup analyses

Boolean variables were compared between brain fog and no brain fog groups using Chi-squared tests of proportions, whilst continuous variables were compared using *t*-tests or Mann Whitney U tests if not normally distributed. Normal distribution was assessed using histograms, Q-Q plots, and Shapiro–Wilk tests of normality. Bonferroni correction for multiple comparisons was used with significance threshold set at 5% (0.05/29 = 0.00172).

The Cochrane-Mantel–Haenszel (CMH) Chi-squared measure was used for the difference between categorical variables and the long-COVID vs. non-long-COVID subgroups, as below.


$$X^{2}=\overset{2}{\underset{i=1}{\sum }}\frac{{\left[\ln\left(OR_{i}\right)-\ln\left(OR_{MH}\right)\right]}^{2}}{\mathrm{var}\left[\ln\left(OR_{i}\right)\right]}$$

MH: Mantel–Haenszel

ANOVA tests were performed to compare continuous variables between the long-COVID and non-long-COVID subgroups.

### Heatmap and clustering

All variables were clustered using agglomerative hierarchical clustering based on their correlations. This method looks at similarity of correlations and can provide useful insights on subjective data. If subjective symptoms cluster, they could be semantically or aetiologically related. We used the exact same method as described elsewhere (Alim-Marvasti et al., 2022a,b).

The method is best applied to continuous variables but may nevertheless provide useful insights with mixed data types. Interpretation should be cautious especially as correlations between Boolean and continuous data are measured differently compared to continuous-continuous data (Jafarzadegan et al., 2019).

### Algorithmic characterization of brain fog

We used the gradient boosted trees algorithm known as XGBoost. XGBoost is an ensemble supervised machine learning method that combines the predictions of multiple weak models (typically decision trees) to create a strong predictive model (Chen and Guestrin, 2016). XGBoost is highly effective for a wide range of machine learning tasks primarily for classification problems and is known for its speed and accuracy.

## Results

25,796 participants used the application, of which 7,280 (28.2%) reported experiencing brain fog. Table 1 shows all the variables.

### Demographics

The mean age of participants was 33.6 ± 11.8 years, and the proportion of females was 89% (*n* = 23,020). People who reported brain fog, were on average 2.9 years older, and more likely to be female (OR = 1.2, *p* < 0.001, Table 1).

Highest level of education was not associated with brain fog (*p* = 0.15).

### Symptoms and functional impairments

Of all symptoms studied, all were statistically significantly associated with brain fog (Table 1). The magnitude of the associations was greatest for difficulty focusing or concentrating (OR = 3.3), difficulty following conversations (OR = 2.2), and difficulty remembering appointments (OR = 1.9).

### Comorbidities and lifestyle factors

The largest associations were with migraine disability scores, long Covid-19, and a history of a diagnosed concussion. The brain fog group scored an average of 34.1% higher on the MIDAS, whilst a history of diagnosed concussions (OR = 2.4, *p* < 0.0001) and long COVID-19 (OR = 3.8, *p* < 0.0001) were significantly more prevalent (Table 1).

Although many other lifestyle factors and comorbidities were associated with brain fog, these were not clinically significant with small magnitudes of association. For example, the single-item sleep quality scale scores sleep from 0 to 10 with 4 to 6 being described as “fair” (Snyder et al., 2018). Although individuals with brain fog reported slightly worse sleep, the difference was 0.3 and both scored in the “fair” region. Similarly, higher anxiety and depression scores, less exercise, and frequent dietary intake of oily fish and nuts and seeds in the diet were all statistically significantly associated with brain fog (all *p* < 0.0001) but their effect sizes were small.

Epilepsy, excess alcohol, and smoking were not associated with brain fog.

### Cognitive scores

Brain fog sufferers scored on average 0.1 standard deviations lower on the M-CogScore test (*p* < 0.0001).

In post-hoc analysis, the three subscores that constitute the M-CogScore, modified Stroop, Symbols, and Memory tests were evaluated (Alim-Marvasti et al., 2022a,b). Amongst the subscores, brain fog sufferers exhibited the largest deficit in M-Stroop scores (0.13 standard deviations lower, *p* < 0.0001) followed by M-Symbols (0.11 standard deviations, *p* < 0.0001). M-Memory, a measure of delayed recall, wasn’t significantly worse in brain fog sufferers after Bonferroni correction (0.06 standard deviations lower in brain fog sufferers, *p* = 0.0018). These cognitive subscore results persisted in the non-long-COVID subgroup but not the long-COVID subgroup (see subgroup analysis below).

### Subgroup analysis: interaction between long-COVID and brain fog

In the subgroup analysis we compared variables in the long-COVID and non-long-COVID subgroups (Supplementary Tables 1, 2). Three categorical variables were significant (*p* < 0.05) before Bonferroni correction: sex, difficulty focusing/concentrating, and difficulty following conversations, and one continuous variable was significant after Bonferroni correction: age.

1. Being male and having had long-COVID was associated with a higher likelihood of reporting brain fog with OR 1.25 in long-COVID and OR 0.82 in non-long-COVID groups (*p* = 0.028).
2. Difficulty focusing or concentrating was more strongly associated with brain fog when experienced in the context of long-COVID (OR 5.54) rather than when experienced without it (OR 3.19, *p* = 0.022).
3. Similarly, difficulty following conversations was more strongly associated with long-COVID OR 2.77 than not with OR 2.14 (*p* = 0.033).

One continuous variable, age, was significant on ANOVA tests even after Bonferroni correction (interaction *p* = 0.0004). Older age in individuals with brain fog was specific to people who had not had long-COVID (non-long-COVID subgroup mean age with brain fog 35.6, mean age without brain fog 32.7, p = 0.0004). That is, people with brain fog and/or long-COVID were older (long-COVID without brain fog 35.3, long-COVID and brain fog 35.8 years).

Besides age, no other variables were significantly different between these two subgroups after Bonferroni correction.

### Subgroup analysis: brain fog and difficulty concentrating

We performed a post-hoc subgroup analysis looking at the minority with brain fog who did not report having difficulty concentrating. From amongst individuals with brain fog, the minority who reported no difficulty concentrating were generally older (39.5 ± 12.8 years vs. 35.4 ± 11.8, *p* < 0.0001), had less functional impairments (difficulty with paperwork, planning, following conversations, remembering appointments, mental arithmetic, and driving; ORs of 0.14, 0.17, 0.27, 0.31, 0.31, and 0.38 respectively, all *p* < 0.0001), lower depression (PHQ no difficulty concentrating 9.341 ± 4.141 vs. difficulty concentrating 14.650 ± 4.858, *p* < 0.0001) and anxiety scores (8.673 ± 4.945 vs. 12.381 ± 4.758), scored lower on the MIDAS scale (6.594 ± 7.230 vs. 12.474 ± 9.428, *p* < 0.0001), reported better sleep (SQS 4.827 ± 2.330 vs. 4.070 ± 2.283), and ate less fast food (Supplementary Table 3). People brain fog and long-COVID were twice as likely to report difficulty concentrating (OR 2.0, *p* = 0.001).

### Heatmap and clustering results

**Figure 1:**
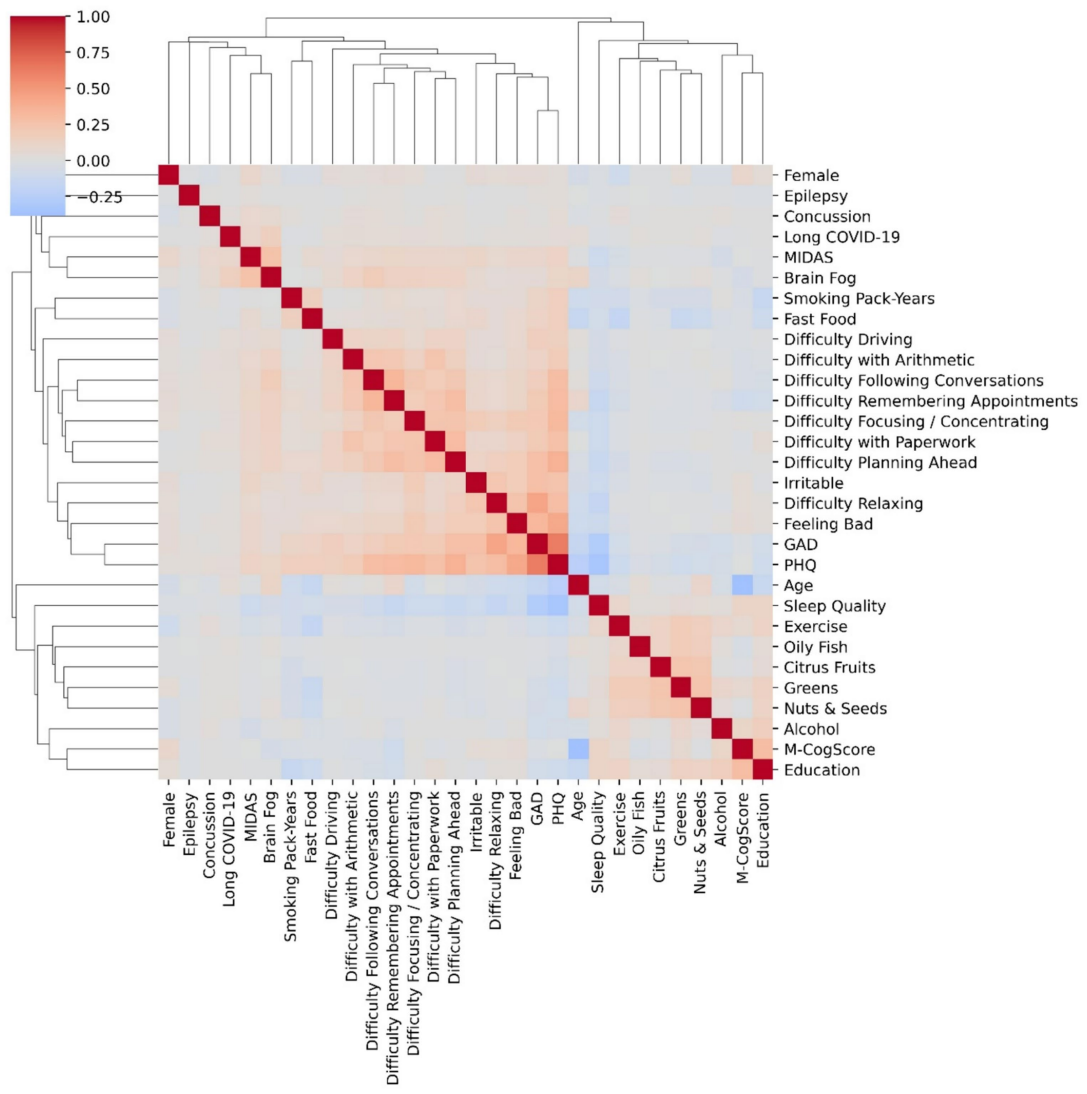
Correlations heatmap of variables and agglomerative hierarchical clustering. Correlations (heatmaps) show degree of correlation: e.g., difficulty relaxing was associated with heightened anxiety scores, and feeling bad was associated with more depression (PHQ). The height of the dendrograms in the clusters determines differences, i.e., the shorter the heights, the more similar the cluster members. Anxiety (GAD score) and depresion (PHQ) were both highly correlated and clustered together with the shortest dendrogram.

Figure 1 shows the correlation heatmap of the variables and that of brain fog, along with the hierarchical clustering dendrogram. The strongest correlations were between anxiety and depression scores, the symptom of difficulty relaxing and anxiety score, and feeling bad and depression (Figure 1). The strongest correlation with brain fog was with the MIDAS score, followed by long COVID-19, concussions, most of the functional difficulties, and inversely correlated with cognitive score (Figure 1).

Hierarchical clustering (Figure 1) suggested that the most similar scores were that of PHQ and GAD, again linking depression and anxiety. There was a separate cluster of diet and sleep scores, except for fast food which clustered with smoking. The M-CogScore cognitive score clustered with highest level of education, whilst brain fog clustered with migraine MIDAS scores, long COVID-19, and concussions. The height of the dendrograms in the clusters determines differences, i.e., the shorter the heights, the more similar the members of the cluster.

### Algorithmic characterization of brain fog

**Figure 2:**
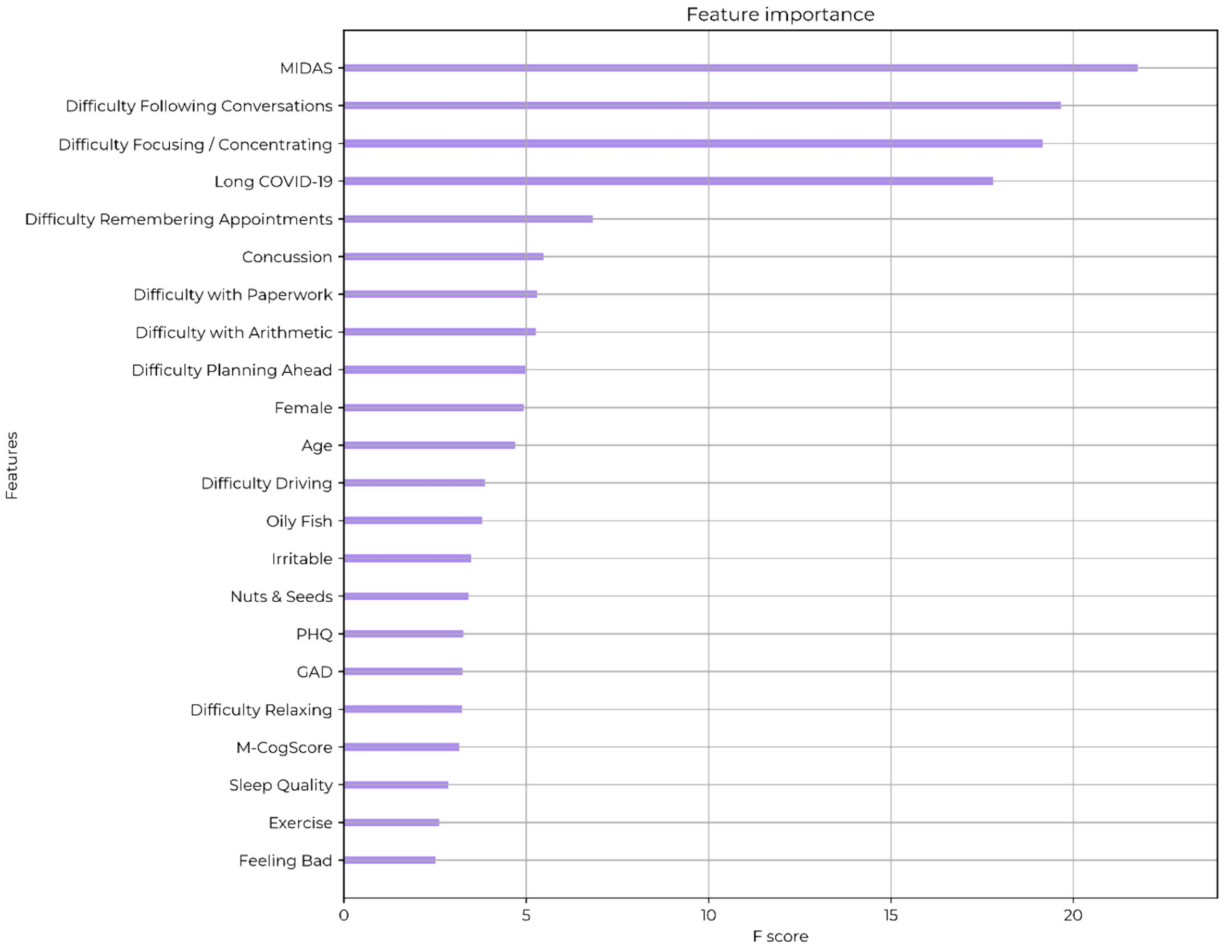
Feature ranking of self-described brain fog. MIDAS: migraine-related functional deficit scores. PHQ: Patient Health Questionnaire (depression score). GAD: generalised anxiety score. M-CogScore: cognitive score combining memory, Stroop, digit-symbols modality test, and speed of test completion.

Extreme gradient boosting algorithms achieved a training accuracy of 85% with cross-validated accuracy of 74% in predicting self-reported brain fog. The feature importance (by gain) for the model are shown in Figure 2. The top symptom was difficulty focusing or concentrating, and the top functional deficit was difficulty following conversations. The most important comorbidities were the MIDAS migraine related score and long COVID-19. The value of an objective cognitive score (M-CogScore) was minimal.

## Discussion

This is the largest study to date looking at the correlates of brain fog in over 25 thousand UK participants. Previous studies used varying definitions of brain fog. Brain fog is, however, by nature, a subjective state and must be allowed to define itself. Brain fog has often been used interchangeably with “mental fog” and “clouding of consciousness.” The term probably originated from the 1817 German equivalent of reduced consciousness “Verdunkelung des Bewusstseins,” which historically has also influenced the definition of delirium (Caraceni and Grassi, 2011). Etymologically, the English term elicits a mental picture of heavy dark clouds over the mind, impairing usual range of foresight, function, and speed.

The strength of this present study lies not only in the large number of participants, but also in its theoretical underpinning of brain fog as primarily a subjective and self-described condition and in characterising it along the four-dimensions of: (1) demographics, (2) symptoms and functional impairments, (3) comorbidities and potential risk factors, and (4) an objective measure of cognition.

Along the demographics axis, people who experienced brain fog were on average 2.9 years (8.8%) older, and more likely to be female (OR = 1.2, *p* < 0.001), congruent previous studies suggesting its association with females with OR 1.4 (Asadi-Pooya et al., 2022). The 8.8% age difference in the on average fourth decade of life is unlikely to represent true mild cognitive impairment or decline, but rather a predisposition to brain fog and its associated risk factors with age. This is also highlighted in the subgroup analysis to dissect difficult differences between long-COVID and brain fog, which showed that the older age in individuals with brain fog was specific to people who had not had long-COVID. Additionally, the relatively young age of participants in this large cohort of over 25 thousand people, with a mean age of 33.6 and standard deviation of 11.8 years, is unlikely to generalise to a significantly older population who will be more likely to have mild cognitive impairment.

Along the symptoms and functional impairments axis, the strongest correlates of brain fog on XGBoost and univariate analyses were difficulty focusing or concentrating (OR = 3.3), following conversations (OR = 2.2), and remembering appointments (OR = 1.9). Additionally, not reporting difficulty concentrating stratified a minority of individuals with brain fog into a less affected group, as they had significantly less functional impairments, better sleep, lower depression and anxiety scores, and lower migraine disability scores.

Along the comorbidities and risk factors axis, all analyses indicated that brain fog was strongly associated with migraine (34.1% higher on the migraine-associated disability scale, MIDAS), a history of long-COVID-19 (OR = 3.8), and a history of previous concussions (OR = 2.4). Brain fog was also more likely to be experienced with poor sleep, anxiety, and depression. Although the anxiety and depression scores were statistically significant, the effect sizes were very small.

The significance of the association with the MIDAS score is that this suggests brain fog sufferers experience symptoms that significantly impair their productivity at home, at work or school, and in social and leisure activities. Future studies should look at broad symptom questionnaires to determine the degree of similarity and differences between the symptoms of migraine and brain fog.

Brain fog has an established association with a wide variety of conditions including migraines, COVID-19, chronic fatigue, insomnia, and systemic infections (Kverno, 2021; Taquet et al., 2021; Jarrott et al., 2022). The strong association we found with long-COVID in all the analyses is therefore expected, especially when considering that the association between brain fog and COVID-19 was widely disseminated and therefore likely influenced internal representations of the identity of the condition, increasing the likelihood of self-reports of brain fog (Leventhal et al., 2020). For example, in widely reported results from a large cohort of over 273,000 individuals and in a systematic review, between 8 and 26% reported impaired concentration and cognitive symptoms from 3 to 6 months post COVID-19 (Michelen et al., 2021; Taquet et al., 2021), in line with the 19% reported elsewhere (Blomberg et al., 2021).

Therefore, hierarchical clustering, univariate analysis, and the XGBoost model, were all in agreement with regards to variables that were most associated with brain fog, confirming previous associations with comorbidities, and further establishing the demographics, symptoms, and self-reported functional impairments of brain fog.

Along the objective cognition axis, we found that brain fog was associated with 0.1 standard deviations lower scores on the M-CogScore scale designed for rapid remote cognitive testing (Alim-Marvasti et al., 2022a,b). This is not unexpected, as the reported symptom of difficulty focusing or concentrating, which was strongly correlated with brain fog, is probably the subjective counterpart to a disturbance in attention and executive functioning (Davis et al., 2021), speed of processing and/or increased cognitive latency (Wells et al., 2020).

On post-hoc analyses of M-CogScore sub-scores, brain fog sufferers showed the largest deficit in the modified Stroop score followed by the modified Digit-Symbols Modality Test score (0.13 and 0.11 standard deviations lower respectively) but did not show statistically significant deficits in delayed recall (Alim-Marvasti et al., 2022a,b). The modified Stroop score is derived using a combination of accuracy and speed over 18 trials (incongruent colours in 12 trials and congruent in six trials) and measures ability to inhibit cognitive interference, selective attention, processing speed, and cognitive flexibility (Stroop, 1935; Scarpina and Tagini, 2017; Alim-Marvasti et al., 2022a,b). In contrast to the M-Symbols and M-Stroop scores, the M-Memory test shows a ceiling effect, which may have limited its sensitivity in detecting a drop in scores (Alim-Marvasti et al., 2022a,b). In contrast, M-Symbols and M-Stroop take account of speed, which can be negatively affected by fatigue. This is consistent with a recent study that demonstrates fatigue being highly associated with brain fog in patients with long covid {Delgado-Alonso et al., 2023 #1947}.

Given the subjective nature of brain fog, and the modest association with cognitive scores here, especially with the modified Stroop, it seems that it cannot be easily measured by a single cognitive sub-domain and does not yet have a suitable objective correlate. It is therefore best characterised subjectively as difficulty focusing and concentrating within the broader context of the four-dimensional characterization and should be differentiated from neurodegenerative conditions and even mild cognitive impairment, even if based on demographics alone. Whilst brain fog is likely to arise from an interaction of physiological, cognitive, and perceptual factors, the molecular and cellular basis of brain fog is yet to be determined. Therefore, it is important to agree as an axiom, that *reversibility* and non-neurodegenerative mechanisms are necessary criteria for a “diagnosis” of brain fog contrary to previous assumptions (Hampshire et al., 2021, Guo et al., 2022). Otherwise, the symptom definition would expand indefinitely and characterisation and interventions will not only be difficult, but also clinically irrelevant (Lebwohl and Ludvigsson, 2014; Lichtwark et al., 2014). Long-COVID-19 sufferers report “good” and “bad” days that do not correlate with objective measures of cognition (Guo et al., 2022). Therefore, brain fog is likely not adequately captured by objective measures alone. We therefore emphasise that brain fog should not be conflated with MCI (Ocon, 2013).

We therefore believe that brain fog is best interpreted subjectively in the context of the above four-dimensions.

Although previous studies have shown that a post COVID-19 cognitive syndrome is associated with changes in brain structure and function, including cognition-impairing grey matter volume loss and reduced functional connectivity (Díez-Cirarda et al., 2023), this has not been shown in non-COVID related brain fog sufferers. Our study suggests that the perception of what constitutes brain fog is universally a difficulty focusing or concentrating, irrespective of a history of long-COVID. As we found no significant differences in brain fog symptoms-axis in the COVID and non-COVID subgroups, brain fog is therefore a rather homogenous subjective experience irrespective of long-COVID status, further supporting its status as a separate entity to neurodegenerative conditions. Our results are in line with multiple other smaller studies that suggest that brain fog is best characterised as difficulty thinking, focusing, communicating, and being forgetful (Ross et al., 2013; Wells et al., 2020; Davis et al., 2021).

### Limitations

A strength and weakness of this study is the use of self-defined brain fog. However, this was collected as a binary variable rather than severity, frequency, and duration of brain fog symptoms. It is also unclear how these results for subjective brain fog would translate in other languages.

Other limitations include that our *a priori* list of variables was not exhaustive. For example, data was not available for infections, coeliac disease, postural orthostatic tachycardia syndrome, chronic fatigue, chronic pain, cancer, use of medications, or chemotherapy (O’Farrell et al., 2013).

We mitigated selection bias by targeting participants seeking to assess their mental and cognitive health using the smartphone application. We mitigated attribution bias of symptoms to brain fog by not directly asking for what participants thought brain fog was and instead used questionnaires on symptoms and comorbidities.

A further limitation is that cognitive function was not comprehensively evaluated using prolonged in-person neuropsychological assessments administered via trained professionals (which remain the gold standard for cognitive evaluations).

Finally, as this was not a longitudinal study, we could not differentiate between potential risk factors and comorbidities and so these were collapsed into one axis.

## Conclusion

This is the largest study of the correlates of subjective brain fog as a syndrome. We posit a four-dimensional characterisation of brain fog based on (1) demographics, (2) symptoms and functional impairments, (3) comorbidities and risk factors, and (4) objective cognitive function.

Brain fog is best characterised subjectively as a difficulty concentrating or focusing that is associated with functional impairments in activities of daily living including difficulty following conversations, remembering appointments, and difficulties with paperwork and mental arithmetic. Brain fog is associated with a history of long-COVID-19, migraines, and concussion, and less so with depression, poorer sleep, and increased anxiety. Brain fog is also associated on average with a 0.1 standard deviation lower objective cognitive score, especially pronounced on modified Stroop testing, suggesting impairments in the ability to inhibit cognitive interference and delayed processing speeds.

Not reporting difficulty concentrating stratified a minority of individuals with brain fog into a less functionally impaired group.

Further studies are required to determine the extent of overlap and differences between the symptoms of brain fog and migraine, given the strength of their association in this study and the prevalence of migraine in the general population.

## Data availability statement

The raw data supporting the conclusions of this article will be made available by the authors, without undue reservation.

## Ethics statement

The studies involving humans were approved by West Midlands research ethics committee. The studies were conducted in accordance with the local legislation and institutional requirements. The participants provided their written informed consent to participate in this study.

## Author contributions

AA-M: Conceptualization, Formal analysis, Funding acquisition, Investigation, Methodology, Resources, Supervision, Validation, Visualization, Writing – original draft, Writing – review & editing. MC: Conceptualization, Investigation, Writing – review & editing. NK: Formal analysis, Investigation, Methodology, Software, Visualization, Writing – review & editing. AL: Data curation, Funding acquisition, Project administration, Resources, Writing – review & editing. HS: Data curation, Funding acquisition, Investigation, Project administration, Resources, Software, Writing – review & editing. MM: Conceptualization, Funding acquisition, Methodology, Resources, Supervision, Validation, Writing – review & editing.
